# Will women outrun men in ultra-marathon road races from 50 km to 1,000 km?

**DOI:** 10.1186/2193-1801-3-97

**Published:** 2014-02-18

**Authors:** Matthias Alexander Zingg, Klaus Karner-Rezek, Thomas Rosemann, Beat Knechtle, Romuald Lepers, Christoph Alexander Rüst

**Affiliations:** Institute of General Practice and for Health Services Research, University of Zurich, Zurich, Switzerland; Gesundheitszentrum St. Gallen, Vadianstrasse 26, 9001 St. Gallen, Switzerland; INSERM U1093, Faculty of Sport Sciences, University of Burgundy, Dijon, France

**Keywords:** Ultra-marathon, Woman, Man, Gender gap, Running speed

## Abstract

It has been assumed that women would be able to outrun men in ultra-marathon running. The present study investigated the sex differences in running speed in ultra-marathons held worldwide from 50 km to 1,000 km. Changes in running speeds and the sex differences in running speeds in the annual fastest finishers in 50 km, 100 km, 200 km and 1,000 km events held worldwide from 1969–2012 were analysed using linear, non-linear and multi-level regression analyses. For the annual fastest and the annual ten fastest finishers, running speeds increased non-linearly in 50 km and 100 km, but not in 200 km and 1,000 km where running speeds remained unchanged for the annual fastest. The sex differences decreased non-linearly in 50 km and 100 km, but not in 200 and 1,000 km where the sex difference remained unchanged for the annual fastest. For the fastest women and men ever, the sex difference in running speed was lowest in 100 km (5.0%) and highest in 50 km (15.4%). For the ten fastest women and men ever, the sex difference was lowest in 100 km (10.0 ± 3.0%) and highest in 200 km (27.3 ± 5.7%). For both the fastest (r^2^ = 0.003, *p* = 0.82) and the ten fastest finishers ever (r^2^ = 0.34, *p* = 0.41) in 50 km, 100 km, 200 km and 1,000 km, we found no correlation between sex difference in performance and running speed. To summarize, the sex differences in running speeds decreased non-linearly in 50 km and 100 km but remained unchanged in 200 km and 1,000 km, and the sex differences in running speeds showed no change with increasing length of the race distance. These findings suggest that it is very unlikely that women will ever outrun men in ultra-marathons held from 50 km to 100 km.

## Background

Ultra-marathon running, *i.e.* running over distances longer than the traditional marathon distance of 42.195 km, is becoming increasingly popular (Hoffman 2010; Hoffman and Krishnan 2013; Hoffman and Wegelin 2009; Zingg et al. 2013a, b, c). Over a hundred thousand of ultra marathoners finish one of more than a thousand races held annually around the world (International Association of Ultrarunners).

In recent years, the growth of ultra-endurance sports has drawn increased attention to investigate trends in both participation and performance of the competitors in ultra-marathon running (Da Fonseca-Engelhardt et al. 2013; Knechtle 2012; Knoth et al. 2012). A major focus of research in endurance sports was the sex difference in performance (Cheuvront et al. 2005; Coast et al. 2004; Lepers and Maffiuletti 2011; Peter et al. 2014; Sparling et al. 1998). Both sexes seemed to have the same age of peak of performance in marathon (Hunter et al. 2011) and over different ultra-marathon distances (Rüst et al. 2013; Zingg et al. 2013a, b), but the question of sex difference in ultra-running performance is still of interest. In elite marathon runners, the sex difference in performance varies across years but has not systemically decreased or varied since the 1980s (Hunter et al. 2011). The comparison of the world best running times from 100 m to 200 km showed that longer distances were associated with greater sex differences with men being ~12.4% faster than women (Coast et al. 2004).

More than two decades ago it was reported in ‘Nature’ that the slope of improvement in the men’s and women’s running records, extrapolated from mean running velocity plotted against historical time, would eventually result in a performance intersection of the sexes across a variety of running distances (Whipp and Ward 1992). It has been suggested that the sex difference in running should disappear with increasing distance, particularly in race distances longer than the marathon (Bam et al. 1997). This suggestion was primarily based on differences in fuel utilization, muscle damage following exercise, relative improvements in performance over the past decades, and on the analysis of marathon *versus* ultra-marathon performances of men and women (Bam et al. 1997). The remaining sex gaps in performance appear biological in origin (Cheuvront et al. 2005). Success in distance running is determined largely by aerobic capacity and muscular strength (Cheuvront et al. 2005). As man have a larger aerobic capacity and a greater muscular strength compared to women, the gap in running performances between men and woman seems unlikely to narrow naturally (Cheuvront et al. 2005). Moreover, running economy at absolute velocities is better in elite male than elite female runners (Daniels and Daniels 1992). However, a potential physiological advantage for women may be a greater fatigue resistance compared to equally trained men in ultra-marathons (Bam et al. 1997).

To evaluate the ongoing question whether women would outrun men in ultra-marathons, a study investigating the sex difference in running performance in ultra-marathons up to ultra-distances of hundreds or even thousands of running kilometres is required. In this context, the aim of the present study was to examine the sex differences in running speeds in ultra-marathons held over different distances from 50 km to 1,000 km. Based upon recent investigations, it was hypothesized that the sex difference in running performance would decrease across years and with increasing length of a race.

## Materials and methods

### Ethics

All procedures used in the study met the ethical standards of the Swiss Academy of Medical Sciences and were approved by the Institutional Review Board of Kanton St. Gallen, Switzerland, with a waiver of the requirement for informed consent of the participants given the fact that the study involved the analysis of publicly available data.

### Data sampling

Race results of all competitors who ever finished a 50 km, 100 km, 200 km and 1,000 km ultra-marathon held worldwide between 1969 and 2012 were collected and analysed. The data set for this study was obtained from the race website of the Deutsche Ultramarathon Vereinigung (DUV). The section http://statistik.d-u-v.org/ records all race results of any ultra-marathon held since 1959 where each competitor is individually recorded with performance, nationality, and age. Data for ultra-marathons held in km seemed not complete before 1969. We therefore recorded for each female and male finisher in a 50 km, 100 km, 200 km and 1,000 km ultra-marathon held worldwide between 1969 and 2012 the race time. The age of the athletes was calculated using the equation calendar year when the race was held – year of birth of the athlete. In total, data from 297,977 finishes (*i.e.* 52,528 female and 245,449 male finishes) were retrieved from the data base. A total of 5,135 female and 22,047 male finishes had to be excluded for data analysis due to missing information about the age of the athletes in the rankings. Finally, complete data with performance and age from 270,795 finishes (*i.e.* 47,393 female and 223,402 male finishes) were included into data analysis.

### Data analysis

To investigate the changes across years in performances and sex differences of the fastest finishers, we restricted to the annual fastest and the annual three fastest women and men. For all regression models, only the top finishers for each year from 1969 to 2012 (43 years) and the top ten men (*i.e.* 43 × 10 = 430) and women (*i.e.* 43 × 10 = 430) were the only cases included in any of the regression models. For each calendar year, the ten fastest races times were sorted for women and men for each ultra-marathon distance (*i.e.* 50 km, 100 km, 200 km and 1,000 km). We determined for the annual fastest and the annual ten fastest women and men the performance (*i.e.* race time) and with these data the sex difference in performance for the annual fastest and the annual ten fastest women and men for each race distance. To determine the sex difference in peak running performance, race times (h) of the annual top and of the annual top ten women and men were determined. To increase the comparability between different race distances regarding performance, all race times were converted to running speed (km/h) using the equation [running speed (km/h)] = [race distance (km)] / [race time (h)]. The sex difference in running speed was calculated using the equation ([running speed in women] – [running speed in men]) / [running speed in men] × 100. The sex difference was calculated for every pair of equally placed athletes (*e.g.* between annual fastest woman and annual fastest men, between annual second fastest woman and annual second fastest men, etc*.*) before calculating mean value and standard deviation of all pairs. When less than the minimal number of athletes was available in a certain year for a certain race distance (*i.e.* a minimum of ten for the annual ten fastest finishers), the calendar year and race times were excluded from data analysis. To find the absolute peak performance and the sex difference in absolute peak performance, the performance of the overall top and overall top ten women and men ever were determined for each ultra-marathon distance and compared to each other. Additionally, the sex differences between the overall top and overall top ten women and men ever were determined and compared as described above. To find a potential relationship between sex difference and performance (*i.e.* running speed), the sex difference in running speed between the top and the top ten women and men ever were compared to the performance of the top and the top ten men for each ultra-marathon distance.

### Statistical analysis

In order to increase the reliability of the data analyses, each set of data was tested for normal distribution and for homogeneity of variances prior to statistical analyses. Normal distribution was tested using a D’Agostino and Pearson omnibus normality test and homogeneity of variances was tested using a Levene’s test. Trends in participation were analysed using regression analysis with ‘straight line’ and ‘exponential growth equation’ model, whereas for each set of data (*e.g.* each sex) both models where compared using Akaike’s Information Criteria (AICc) to decide which model showed the highest probability of correctness. Single and multi-level regression analyses were used to investigate the changes in running speed and sex difference in running speed. A hierarchical regression model was used for the analysis of the annual top and the annual top ten athletes to avoid the impact of a cluster-effect on the results in case one athlete finished more than once in the annual top or the annual top ten. Since the change in the differences in the performance between the sexes is assumed to be non-linear (Reinboud 2004), we additionally calculated the non-linear regression models that fit the data best. We compared the best-fit non-linear models to the linear models using Akaike’s Information Criteria (AIC) and F-test in order to show which model (*i.e.* non-linear *versus* linear) would be the most appropriate to explain the trend of the data. To compare performance and sex difference between multiple groups (*e.g.* men *versus* women or between different race distances), a one-way analysis of variance (ANOVA) with subsequent Tukey-Kramer post-hoc analysis (*i.e.* one family per row in case of two-dimensionally array of data) was used. A potential relationship between the sex difference of the fastest runners and running speed was investigated using correlation analysis. The sex differences between the top and the top ten women and men ever were compared to the performance of men to find a potential relationship between sex difference and running speed. Statistical analyses were performed using IBM SPSS Statistics (Version 21, IBM SPSS, Chicago, IL, USA) and GraphPad Prism (Version 6.01, GraphPad Software, La Jolla, CA, USA). Significance was accepted at *p* < 0.05 (two-sided for *t*-tests). Data in the text and figures are given as mean ± standard deviation (SD).

## Results

### Participation trends

**Figure 1:**
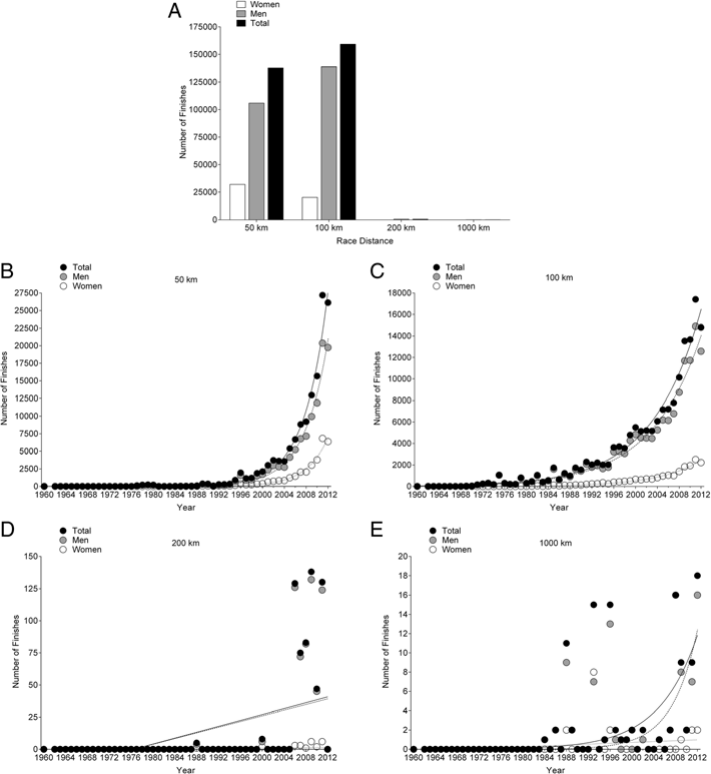
**Number of events (Panel A) and finishes in 50 km (Panel B), 100 km (Panel C), 200 km (Panel D) and 1,000 km (Panel E) from 1969–2012.**

Most of the competitors finished a 100 km, followed by finishers in a 50 km (Figure 1A). Of overall finishers from 50 km to 1,000 km, 17.6% were women and 82.4% were men. The percentage of female finishers decreased from 50 km (23.2%) to 100 km (12.8%) and to 200 km (3.9%) but was relatively high in 1,000 km (17.6%). The number of finishes increased exponentially in 50 km (Figure 1B), 100 km (Figure 1C) and 1,000 km (Figure 1E), but only linearly in 200 km (Figure 1D).

### Changes in running speeds across years

**Figure 2:**
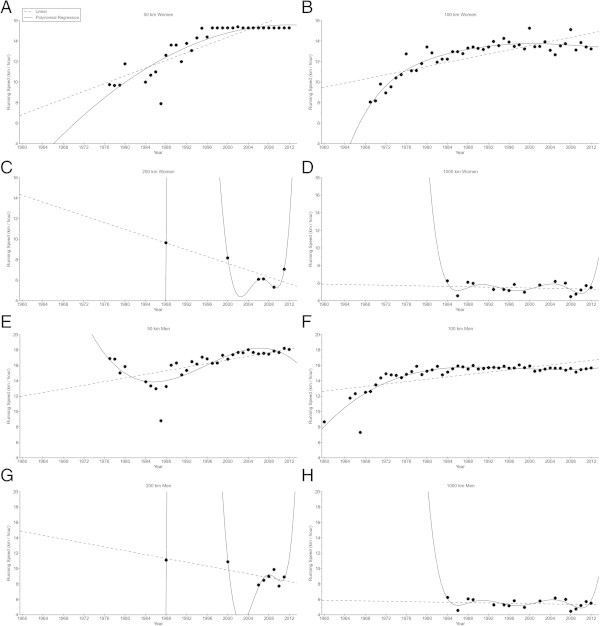
**Running speeds of the annual fastest women in 50 km (Panel A), 100 km (Panel B), 200 km (Panel C) and 1,000 km (Panel D) and for the annual fastest men in 50 km (Panel E), 100 km (Panel F), 200 km (Panel G) and 1,000 km (Panel H).**

Figure 2 presents the changes in running speeds across years for the annual fastest female and male runners in 50 km, 100 km, 200 km, and 1,000 km. For both women and men, the annual fastest finishers improved in 50 km and 100 km, but not in 200 km and 1,000 km, also when corrected for multiple finishes (Table 1). For the annual fastest men, running speed increased non-linearly in 50 km from 16.88 km/h (1977) to 18.06 km/h (2012) (*i.e.* polynomial regression 3rd degree) and non-linearly in 100 km from 8.67 km/h (1960) to 15.65 km/h (2012) (*i.e.* polynomial regression 4th degree) (Table 2). In 200 km and 1,000 km, running speeds remained unchanged at 9.23 ± 1.28 km/h and 5.47 ± 0.56 km/h, respectively. In women, running speeds of the annual fastest finishers increased non-linearly in 50 km from 9.74 km/h (1977) to 15.28 km/h (2012) (*i.e.* polynomial regression 2nd degree) and non-linearly in 100 km from 8.06 km/h (1969) to 13.22 km/h (2012) (*i.e.* polynomial regression 6th degree) (Table 2). In 200 km and 1,000 km, running speeds remained unchanged at 7.09 ± 1.27 km/h and 4.52 ± 0.60 km/h, respectively.

**Table 1:** **Multi-level regression analyses for changes in running speeds across years for the annual fastest and the annual ten fastest female and male runners (Model 1) with correction for multiple finishes (Model 2) and with correction for multiple finishes and age of athletes with multiple finishes (Model 3)**

Distance	Model	***β***	SE ( ***β*** )	Stand. ***β***	T	***P***
**Annual fastest men**						
**50 km**	1	0.118	0.027	0.614	4.333	< 0.0001
	2	0.118	0.027	0.614	4.333	< 0.0001
	3	0.105	0.028	0.547	3.816	< 0.0001
**100 km**	1	0.077	0.014	0.640	5.705	< 0.0001
	2	0.077	0.014	0.640	5.705	< 0.0001
	3	0.084	0.014	0.696	5.953	< 0.0001
**200 km**	1	-0.125	0.046	-0.742	-2.711	0.035
	2	-0.125	0.046	-0.742	-2.711	0.035
	3	-0.125	0.050	-0.741	-2.469	0.057
**1,000 km**	1	-0.011	0.015	-0.181	-0.711	0.488
	2	-0.011	0.015	-0.181	-0.711	0.488
	3	-0.013	0.016	-0.217	-0.822	0.425
**Annual fastest women**						
**50 km**	1	0.188	0.020	0.857	9.246	< 0.0001
	2	0.188	0.020	0.857	9.246	< 0.0001
	3	0.191	0.024	0.870	7.867	< 0.0001
**100 km**	1	0.101	0.013	0.780	8.068	< 0.0001
	2	0.101	0.013	0.780	8.068	< 0.0001
	3	0.095	0.009	0.728	10.850	< 0.0001
**200 km**	1	-0.166	0.045	-0.878	-3.663	0.022
	2	-0.166	0.045	-0.878	-3.663	0.022
	3	-0.168	0.062	-0.892	-2.701	0.074
**1,000 km**	1	-0.042	0.021	-0.580	-2.016	0.079
	2	-0.042	0.021	-0.580	-2.016	0.079
	3	-0.046	0.022	-0.636	-2.084	0.076
**Annual ten fastest men**						
**50 km**	1	0.155	0.009	0.707	18.123	< 0.0001
	2	0.155	0.009	0.707	18.123	< 0.0001
	3	0.145	0.009	0.663	16.966	< 0.0001
**100 km**	1	0.063	0.003	0.656	18.195	< 0.0001
	2	0.063	0.003	0.656	18.195	< 0.0001
	3	0.063	0.003	0.663	18.365	< 0.0001
**200 km**	1	0.007	0.069	0.013	0.102	0.919
	2	0.007	0.069	0.013	0.102	0.919
	3	0.036	0.069	0.068	0.517	0.607
**1,000 km**	1	-0.027	0.014	-0.334	-1.877	0.071
	2	-0.027	0.014	-0.334	-1.877	0.071
	3	-0.026	0.015	-0.321	-1.759	0.090
**Annual ten fastest women**						
**50 km**	1	0.176	0.008	0.822	22.702	< 0.0001
	2	0.176	0.008	0.822	22.702	< 0.0001
	3	0.178	0.008	0.828	22.229	< 0.0001
**100 km**	1	0.091	0.005	0.670	16.845	< 0.0001
	2	0.091	0.005	0.670	16.845	< 0.0001
	3	0.091	0.005	0.672	17.598	< 0.0001

**Table 2:** **Comparison of linear and non-linear regression analysis of changes in running speeds across years in the annual fastest women and men to determine which model is the best**

Running speed	Kind of regression	Sum of squares	DOF	AICc	Best regression	Best regression	Delta	Probability	Likelihood
					AIC-Test	F-Test			
Annual fastest men 50 km	Polynomial	45.22	29	17.22	Polynomial	Polynomial	12.99	0.0015	99.8%
	Linear	77.31	31	30.22					
Annual fastest women 50 km	Polynomial	33.46	30	4.86	Polynomial	Polynomial	6.30	0.041	95.9%
	Linear	43.38	31	11.16					
Annual fastest men 100 km	Polynomial	29.45	44	-16.02	Polynomial	Polynomial	45.56	1.27 e^-10^	100%
	Linear	85.81	47	29.54					
Annual fastest women 100 km	Polynomial	14.83	37	-33.57	Polynomial	Polynomial	38.62	4.10 e ^-09^	100%
	Linear	47.05	42	5.04					
Annual fastest men 200 km	Polynomial	3.30	0	4.15	Linear	Undetermined	22.70	1.17 e ^-05^	99.9%
	Linear	4.99	15	-18.54					
Annual fastest women 200 km	Polynomial	0.011	0	-27.78	Polynomial	Undetermined	26.48	1.77 e ^-06^	99.9%
	Linear	2.92	4	-1.30					
Annual fastest men 1,000 km	Polynomial	1.65	0	1.39	Linear	Undetermined	2.24	0.24	75.4%
	Linear	5.15	6	-0.84					
Annual fastest women 1,000 km	Polynomial	1.08	0	-4.19	Linear	Undetermined	9.72	0.0076	99.2%
	Linear	1.93	8	-13.92					

**Figure 3:**
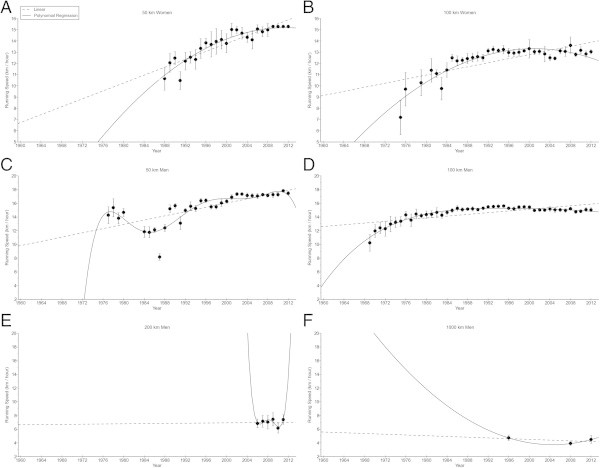
**Running speeds of the annual ten fastest women in 50 km (Panel A) and 100 km (Panel B) and annual ten fastest men in 50 km (Panel C), 100 km (Panel D), 200 km (Panel E) and 1,000 km (Panel F).**

Also for the annual ten fastest finishers (Figure 3), both women and men improved in 50 km and 100 km, also when corrected for multiple finishes (Table 1). For the annual ten fastest men, running speed increased linearly in 50 km from 14.2 ± 1.2 km/h (1977) to 17.5 ± 0.2 km/h (2012) and non-linearly in 100 km from 10.2 ± 1.2 km/h (1969) to 15.0 ± 0.3 km/h (2012) (*i.e.* polynomial regression 4th degree) (Table 3). In 200 km and 1,000 km, running speed remained unchanged at 7.0 ± 0.47 km/h and 4.36 ± 0.42 km/h, respectively. In women, running speed of the annual ten fastest finishers increased non-linearly from 10.6 ± 1.0 km/h (1988) to 15.3 ± 0.0 km/h (2012) in 50 km (*i.e.* polynomial regression 2nd degree) and non-linearly from 7.2 ± 1.5 km/h (1975) to 13.0 ± 0.2 km/h (2012) in 100 km (*i.e.* polynomial regression 2nd degree) (Table 3).

**Table 3:** **Comparison of linear and non-linear regression analysis of changes in running speeds across years in the annual ten fastest women and men to determine which model is the best**

Running speed	Kind of regression	Sum of squares	DOF	AICc	Best regression	Best regression	Delta	Probability	Likelihood
					AIC-Test	F-Test			
Annual ten fastest men 50 km	Polynomial	29.00	16	61.73	Linear	Linear	33.98	4.16 e^-08^	100%
	linear	71.73	31	27.75					
Annual ten fastest women 50 km	Polynomial	5.87	22	-31.64	Polynomial	Polynomial	8.34	0.015	98.5%
	Linear	9.02	23	-23.30					
Annual ten fastest men 100 km	Polynomial	3.08	39	-107.90	Polynomial	Polynomial	88.13	7.28 e^-20^	100%
	Linear	26.76	42	-19.77					
Annual ten fastest women 100 km	Polynomial	9.57	32	-41.00	Polynomial	Polynomial	34.71	2.89 e^-08^	100%
	Linear	27.52	33	-6.29					
Annual ten fastest men 200 km	Polynomial	0.36	0	-6.75	Linear	Undetermined	0.27	0.46	53.4%
	Linear	1.12	4	-7.02					
Annual ten fastest women 1,000 km	Polynomial	1.32 e^-20^	0	-136.61	Polynomial	Undetermined	135.24	4.27 e^-30^	100%
	Linear	0.25	1	-1.36					

### Changes in sex differences in running speeds across years

**Figure 4:**
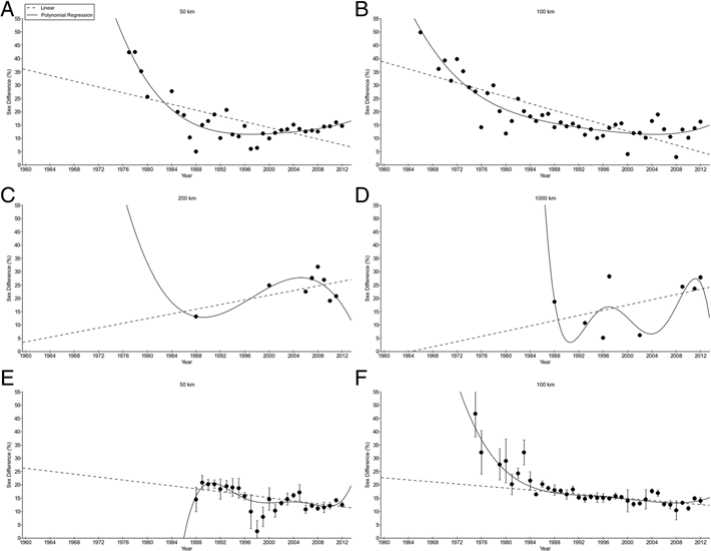
**Sex differences of the annual fastest finishers in 50 km (Panel A), 100 km (Panel B), 200 km (Panel C) and 1,000 km (Panel D) and the annual ten fastest finishers in 50 km (Panel E) and 100 km (Panel F).**

Figure 4 presents the changes in sex differences in running speeds for the annual fastest finishers for 50 km (Figure 4A), 100 km (Figure 4B), 200 km (Figure 4C) and 1,000 km (Figure 4D). For the annual ten fastest finishers, only athletes for 50 km (Figure 4E) and 100 km (Figure 4F) could be considered. The sex differences decreased across years in 50 km and 100 km, but not in 200 and 1,000 km, also when controlled for multiple finishes (Table 4). In 50 km, the sex difference decreased non-linearly for the fastest finishers from 42.3% (1977) to 14.6% (2102) (*i.e.* polynomial regression 4th degree) (Table 5). Also in 100 km, the sex difference decreased non-linearly for the fastest finishers from 56.1% (1965) to 16.3% (2012) (*i.e.* polynomial regression 2nd degree) (Table 5). In 200 km and 1,000 km, the sex differences remained unchanged for the fastest finishers at 23.4 ± 5.6% and 18.1 ± 9.5%, respectively.

**Table 4:** **Multi-level regression analyses for the changes in sex differences across years for the annual fastest and the annual ten fastest runners (Model 1) and with correction for multiple finishes (Model 2)**

Distance (km)	Model	***β***	SE ( ***β*** )	Stand. ***β***	T	***P***
**Annual fastest athletes**						
**50**	1	-0.545	0.124	-0.620	-4.396	< 0.0001
	2	-0.545	0.124	-0.620	-4.396	< 0.0001
**100**	1	-0.652	0.076	-0.789	-8.526	< 0.0001
	2	-0.652	0.076	-0.789	-8.526	< 0.0001
**200**	1	0.439	0.254	0.576	1.724	0.135
	2	0.439	0.254	0.576	1.724	0.135
**1,000**	1	0.493	0.386	0.463	1.278	0.249
	2	0.493	0.386	0.463	1.278	0.249
**Annual ten fastest athletes**						
**50**	1	-0.293	0.041	-0.416	-7.199	< 0.0001
	2	-0.293	0.041	-0.416	-7.199	< 0.0001
**100**	1	-0.526	0.027	-0.714	-19.295	< 0.0001
	2	-0.526	0.027	-0.714	-19.295	< 0.0001

**Table 5:** **Comparison of linear and non-linear regression analysis of changes in sex differences across years in the annual fastest and the annual ten fastest to determine which model is the best**

Sex difference	Kind of regression	Sum of squares	DOF	AICc	Best regression	Best regression	Delta	Probability	Likelihood
					AIC-Test	F-Test			
Annual fastest 50 km	Polynomial	444.34	28	95.23	Polynomial	Polynomial	35.10	2.38 e^-08^	100%
	Linear	1606.07	31	130.33					
Annual fastest 100 km	Polynomial	728.25	41	136.02	Polynomial	Polynomial	42.52	5.82 e^-10^	100%
	Linear	2132.06	44	178.55					
Annual fastest 200 km	Polynomial	77.27	4	30.14	Linear	Linear	3.64	0.13	86.1%
	Linear	157.23	6	26.49					
Annual fastest 1,000 km	Polynomial	291.95	0	42.77	Linear	Undetermined	7.02	0.028	97.1%
	Linear	500.11	6	35.74					
Annual ten fastest 50 km	Polynomial	181.77	19	62.75	Polynomial	Polynomial	5.57	0.058	94.2%
	Linear	352.50	23	68.32					
Annual ten fastest 100 km	Polynomial	255.72	30	82.58	Polynomial	Polynomial	28.31	7.08 e^-07^	99.9%
	Linear	738.96	34	110.9					

For the annual ten fastest finishers, the sex difference decreased non-linearly in 50 km between 1988 and 2012 from 14.6 ± 4.6% to 12.6 ± 1.0% (*i.e.* polynomial regression 5th degree) (Table 5). Also for the annual ten fastest 100 km ultra-marathoners, the sex difference decreased non-linearly across years from 46.7 ± 8.7% (1975) to 14.0 ± 1.2% (2012) (*i.e.* polynomial regression 5th degree) (Table 5).

### Running performances and sex differences for the fastest finishers ever

**Figure 5:**
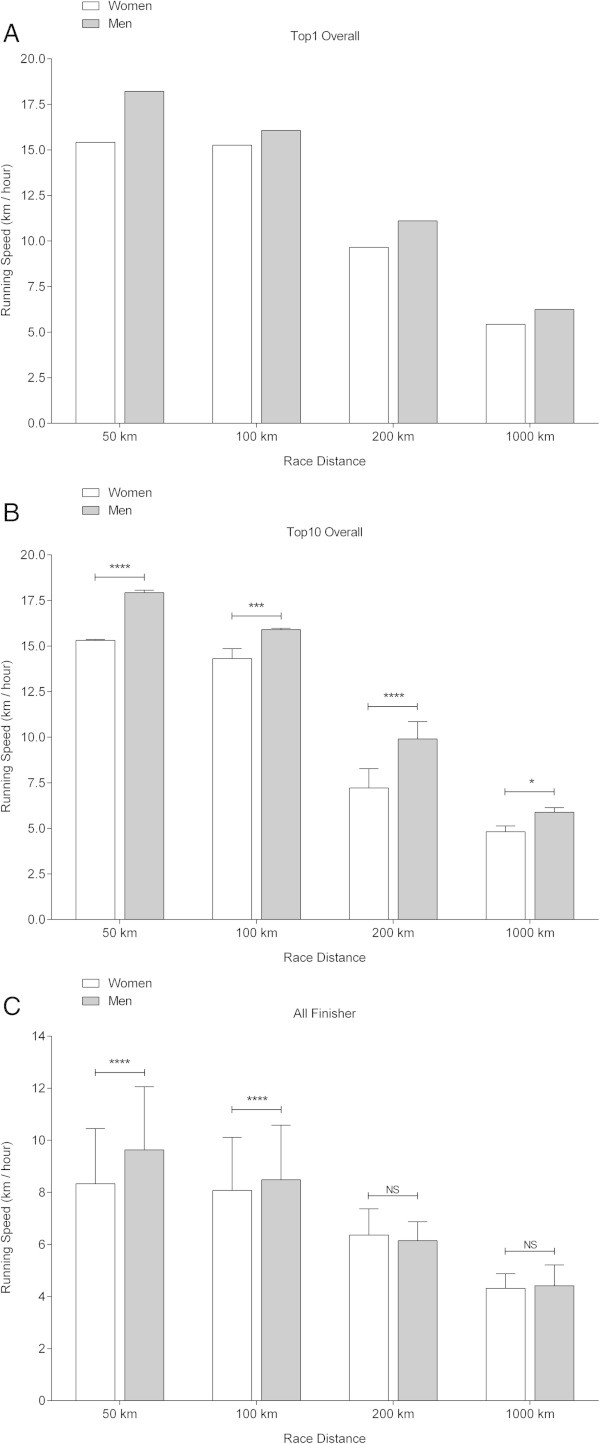
**Running speeds of the fastest finishers ever (Panel A), the ten fastest finishers ever (Panel B) and all finishers ever (Panel C).** NS = not significant; * = *p* < 0.05, ** = *p* < 0.01, *** = *p* < 0.001, **** = *p* < 0.0001).

Figure 5 presents the running speeds of the fastest women and men ever (Figure 5A), the ten fastest women and men ever (Figure 5B) and for all finishers ever (Figure 5C). In the fastest women and men ever and the ten fastest women and men ever, the fastest men were always faster than the fastest women for all distances from 50 km to 1,000 km. Regarding overall finishers, men were only faster than women for 50 km and 100 km, but not for 200 km and 1,000 km.

**Figure 6:**
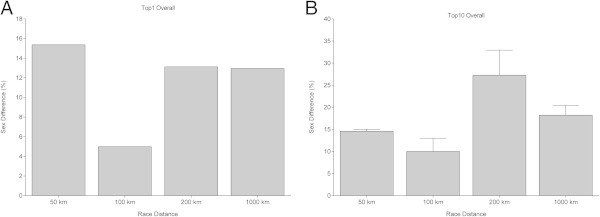
**Sex differences of the fastest finishers ever (Panel A) and the ten fastest finishers ever (Panel B).**

In Figure 6, the sex differences for the fastest ever (Figure 6A) and the ten fastest ever (Figure 6B) are presented. The sex difference in running speed for the fastest women and men ever was lowest in 100 km (5.0%) and highest in 50 km (15.4%). When the fastest ten women and men were considered, the sex difference was lowest in 100 km (10.0 ± 3.0%) and highest in 200 km (27.3 ± 5.7%).

**Figure 7:**
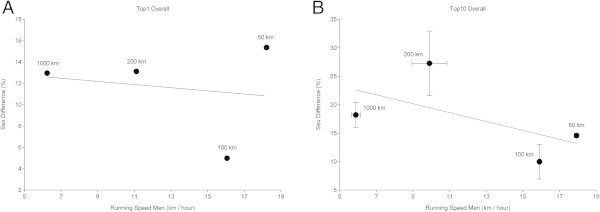
**Correlation between sex difference and running speed of the fastest men ever (Panel A) and the ten fastest men ever (Panel B).**

Figure 7 presents the correlation between sex differences and running speeds in men. The sex differences between the top (Figure 7A) and the top ten (Figure 7B) women and men ever were compared to the performance of men to find a potential relationship between sex difference and running performance. For both the fastest finishers ever (r^2^ = 0.003, *p* = 0.82) and the ten fastest finishers ever (r^2^ = 0.34, *p* = 0.41), we found no correlation between sex difference and running speed.

## Discussion

This study examined the sex differences in running speeds in ultra-marathons held worldwide from 50 km to 1,000 km and it was hypothesized that the sex differences would decrease across years and with increasing length of a race. The main findings were for both women and men that (*i*) the fastest finishers improved their running speeds across years in 50 km and 100 km, but not in 200 km and 1,000 km, (*ii*) the sex differences in running speeds decreased non-linearly in 50 km and 100 km, but not in 200 km and 1,000 km and (*iii*) the sex differences showed no changes with increasing length of the race distance. These findings suggest that it is very unlikely that women will ever outrun men in ultra-marathons held from 50 km to 1,000 km.

### Participation trends

A first important finding was that 100 km ultra-marathons were the most popular races, followed by 50 km events. In contrast to the assumption that the numbers of finishers would decrease with increasing distance, competing in a 100 km ultra-marathon was more popular than in a 50 km ultra-marathon. A possible explanation might be the special number of ‘one hundred kilometre’ attracting ultra-marathoners more than any other ultra-marathon distance. A similar finding was reported by Teutsch et al. (2013) investigating 24 hour and 12 hour ultra-marathons where more athletes competed in the 24 hour than in the 12 hour race. Another explanation could be the fact that 50 km is only a little longer than the classical marathon distance. A runner may therefore prefer running the well-known marathon than the rather unknown 50 km ultra-marathon.

On the other side, the increase in the number of finishes across all distances was not surprising. As the first ultra-marathons were held in the 70’s (International Association of Ultrarunners), an increase in the number of finishers was to be expected across years. Since more 100 km races were held worldwide than any other ultra-marathon distance from 50 km to 1,000 km, consequently more runners finished a 100 km than any other ultra-marathon (International Association of Ultrarunners).

An increase in the number of finishers in ultra-marathons has already been reported (Hoffman and Wegelin 2009; Knoth et al. 2012; Teutsch et al. 2013). An analysis of participation in 161 km ultra-marathons held in North America showed that the number of both competitions and competitors significantly increased over the last decades (Hoffman and Wegelin 2009). Whereas other studies reported mainly numbers of single events, our data confirm an increase in the numbers of finishers in ultra-endurance running races held worldwide. Considering the numbers of finishers, the percentage of female finishers increased in both 50 km (23.2% in 2012) and 100 km (12.8% in 2012). An increase in female participation has been reported since 1977 in the ‘Western States 100-Mile Endurance Run’ in the USA (Hoffman and Wegelin 2009). The percentage of female athletes increased from 10% to 20% in the late 1980s to 20% to 22% since 2001.

### Changes in running speeds across years

Another important finding was that for both women and men, the annual fastest finishers improved their performance in 50 km and 100 km, but not in 200 km and 1,000 km. Also for the annual ten fastest finishers, both women and men improved in 50 km and 100 km. These trends were in line with the findings of Rüst et al. (2013) investigating performance trends in 100miles runners and showing an increase in running speed by 13.7% for women and by 14.5% for men for the annual ten fastest runners from 1998 to 2011. In distances longer than 200 km, Zingg et al. (2013a) reported an increase in running speed from 2000 to 2012 in the 217 km ‘Badwater’ from 7.9 ± 0.7 km/h to 8.7 ± 0.6 km/h (+10.1%) for men and from 5.4 ± 1.1 km/h to 6.6 ± 0.5 km/h (+22.2%) for women. Therefore, running performance still improves in ultra-marathons whereas in running up to marathon distance, improvements are accomplished at a much lower rate.

A possible explanation for this finding may be explained by economic reasons. Marathon running has become a lucrative sport in recent years (World Marathon Majors 2013). Ultra-marathon running, however, is still predominantly non-professional for both elite and recreational runners (Hoffman and Fogard 2012). Another reason for the improvement in the annual fastest finishers could be explained by the increasing number of finishers. It may be argued that an increasing number of finishers increased the density of elite runners. Our data support this assumption as not only the fastest but also the annual ten fastest runners improved their performance across years. The question where the limits in running speed over these ultra-distances may be found cannot be answered so far.

### Sex differences in running performances

Regarding the changes in sex differences across years, previous studies suggested a decrease in sex differences and a stabilization afterwards (Coast et al. 2004; Zingg et al. 2013b). While the sex differences in running speeds decreased in the fastest finishers in both 50 km and 100 km, the sex differences remained unchanged in 200 km and 1,000 km. In contrast to 50 km and 100 km races, the 200 km and 1,000 km events were rarely held and had only a few finishers. The non-linear decreases in sex differences in 50 km and 100 km and the unchanged sex differences in 200 km and 1,000 km suggest that women will not outrun men in ultra-marathons held from 50 km to 1,000 km.

A further finding was that for all distances from 50 km to 1,000 km men were faster than women regarding the fastest ever, the ten fastest ever, the annual fastest and the annual ten fastest finishers. However, considering all finishers for all distances men were only faster in 50 km and 100 km, but not in 200 km and 1,000 km. This finding is in line with the observation of Bam et al. (1997) for ultra-marathons up to 90 km. It seemed that the sex difference of overall women’s and men’s running speed disappears as race distance increases. A possible explanation may be that only the very fittest women participate in ultra-marathons, especially in very long ultra-marathons of 200 km and more whereas not only the fittest men but also strong motivated recreational male athletes compete. A seemingly paradox finding was that the percentage of female finishers decreased with increasing race distance which was previously reported for runners competing in ultra-marathons over all distances held in miles (Zingg et al. 2013b).

Regarding peak running speed, the sex difference has been investigated for both elite and recreational runners (Coast et al. 2004). Coast et al. (2004) compared the world best running times at distances from 100 m to 200 km and found that men were ~12.4% faster than women. Medic et al. (2009) reported sex differences in performance in different sports such as swimming and track or field running and found them to be quite constant at ~10%. In running, it seemed that the sex difference in performance in the fastest finishers increased with increasing distance (Coast et al. 2004). A number of authors published data concerning sex differences in running speed in marathons (Coast et al. 2004; Hunter and Stevens 2013) and ultra-marathons (Hoffman 2008; Hoffman 2010; Hoffman and Wegelin 2009; Zingg et al. 2013a, b, c). In the top ten finishers in the 78-km ‘Swiss Alpine’ a decrease of the sex difference in running speed was reported from 22% (1998) to 17% (2012) (Eichenberger et al. 2012). Over longer distances such as the 100miles in the ‘Western States Endurance Run’, Hoffman and Wegelin (2009) found a sex difference of an average 20% from 1989–2008. In the 217-km ‘Badwater’ a sex difference of 19.8% ± 4.8% and in the 246-km ‘Spartathlon’ of 19.6% ± 2.5% were reported (Zingg et al. 2013a). In the present data, however, in the fastest ever and ten fastest ever, the sex difference was lowest in 100 km and highest in 50 km for the fastest men and woman and in 200 km for the ten fastest men and woman. Thus, no systematic trend could be observed in the present investigation. As sex difference in performance showed no change with increasing distance, women will most probably not outrun men in any ultra-marathon distance between 50 km and 1,000 km.

### Strength and limitations of the study

The main strength of our investigation is the large sample size. However, variables such as anthropometry (Knechtle et al. 2012), physiology (Murray and Costa 2012), previous experience (Hoffman and Krishnan 2013), training (Rüst et al. 2012), psychological considerations (Krouse et al. 2011), nutrition (Machefer et al. 2007), and nationality (Cejka et al. 2013) were not considered. This might have had an influence on the results.

## Conclusion

In summary, the sex differences in running speeds decreased non-linearly in 50 km and 100 km but remained unchanged in 200 km and 1,000 km. Additionally, the sex differences in running performances showed no change with increasing length of the race distance. These findings suggest that it is very unlikely that women will outrun men ever in ultra-marathons held from 50 km to 100 km.
